# Optimization of COD, nitrate-N and phosphorus removal from hatchery wastewater with acclimatized mixed culture

**DOI:** 10.1016/j.heliyon.2022.e09217

**Published:** 2022-03-29

**Authors:** Norazwina Zainol, Kamaliah Abdul Samad, Che Asilah Ilyana Che Jazlan, Nurul Ain Razahazizi

**Affiliations:** a College of Engineering, Universiti Malaysia Pahang, Lebuhraya Tun Razak, 26300 Gambang, Kuantan, Pahang, Malaysia; b Earth Resources and Sustainability Centre (ERAS), Universiti Malaysia Pahang, 26300 Gambang, Kuantan, Pahang, Malaysia; c Faculty of Chemical and Process Engineering Technology, Universiti Malaysia Pahang, Lebuhraya Tun Razak, 26300 Gambang, Kuantan, Pahang, Malaysia

**Keywords:** Chemical oxygen demand, Nitrate-N, Phosphorus, Acclimatized mixed culture, Hatchery wastewater

## Abstract

The goal of this study is to optimize the condition of the pollutant removal process by using acclimatized mixed culture (AMC) in the treatment of contaminated waste from the hatchery industry. The removal of chemical oxygen demand (COD), nitrate-N, and total phosphorus was optimized using a central composite design and the Response Surface Methodology (RSM) with two parameters: AMC content and retention time (days). Each factor had a range value of 15%–35% AMC content and a retention time of 3–5 days, with COD, nitrate-N, and total phosphorus removal as responses. Prior to experimentation, the synthetic wastewater was prepared, and the mixed cultures were acclimatized. In 13 runs, the experiment was carried out in accordance with the setup created by the Design-Expert software. The sample was tested for COD, nitrate-N, and total phosphorus using a Hach spectrophotometer. The findings show a strong relationship between predicted and experimental COD, nitrate-N, and total phosphorus removal values. At optimum conditions of 29% AMC content and 4 days of retention time, removal of COD, nitrate-N, and total phosphorus was observed to be 28%, 80% and 36%, respectively. The discovery also revealed that maximum values of removal of 62% COD, 94% nitrate-N, and 46% total phosphorus could be obtained under various optimum conditions. The study shows that, the acclimatized mixed culture (AMC) can be used as a potential biological wastewater treatment as well as a natural removal of COD, nitrate-N, and total phosphorus.

## 1 Introduction

Hatcheries are critical components of the aquaculture system because they are designed and operated specifically for the purpose of raising larvae economically [1]. Yet there are many challenges faced by the industry around the world which among that is finding a way to provide a hatchery environment that is best for larval growth without negatively impacting production economics [2]. The poor quality of water is the most common stressor present in the hatchery [2]. The high amounts of wastewater containing compounds including total nitrogen and total phosphorus were the main obstacles for hatchery wastewater [3]. The main source of nitrogen and phosphorus is derived from feed application, where a large fraction of it will remain as uneaten food and metabolic waste in the pond [4].

Shrimps are one of the most fundamental commodities of the global fishery trade [5] that has experienced significant expansion and contributes significantly to worldwide aquaculture productivity. However, the rise of shrimp aquaculture has had unforeseen repercussions, such as wastewater management challenges and other concerns related to the environmental impact of the effluent. Most of the nutrients used in shrimp aquaculture are obtained from outside sources such as on-farm formulated feeds and commercially available fertilizers. Shrimp feed that is not consumed or digested accumulates at the bottom of the pond and is destroyed by microbes [6]. The biological oxygen demand (BOD) of the pond water is increased as a result of this activity. To increase the primary productivity of shrimp ponds, fertilizers are added [7]. Yet, shrimp feeds and fertilizers have higher quantities of nitrogen and phosphorus than the water used in the cultivation process. Nitrogen and phosphorus are the primary constituents that cause eutrophication in aquaculture wastewater, which leads to the destruction of the ecosystem. Therefore, proper effluent treatment prior to discharge into water bodies is critical, because the sustainability of shrimp hatcheries and appropriate handling are dependent on the availability of a clean water source in continuity [8].

The need to abide by the current National Pollution Discharge Elimination System (NPDES) authorising measures has inducted the thought of substitutive treatment techniques for hatchery wastewater. The potential pollution problems for regular hatchery effluent include oxygen depletion, nutrient enrichment, taste and odour in cases where the receiving water flow is low. Conventional wastewater treatment used in aqua culturing systems relies on a variety of biological processes [3]. In the hatchery industry, mixed culture is one of the methods that are used in biological treatment to treat the wastewater. The advantages of applying mixed culture include reducing the levels of ammonia, nitrite, nitrate, and phosphorus, increasing the concentrations of dissolved oxygen, and promoting the decomposition of organic matter [9]. Additionally, mixed culture is capable of absorbing soluble organic compounds directly, which consequently contributed to the removal of chemical oxygen demand removal (COD) [10].

Nutrients may be removed chemically, physically, or biologically in conventional wastewater treatment. Of all the methods, the biological method seems to be the most efficient due to its cost-efficiency, as less energy consumption and maintenance requirements are needed [11]. The application of microbials in wastewater treatment has been widely used. The anaerobic-anoxic-aerobic method (A2O method) is one of the most effective methods for removing nitrogen and phosphorus from wastewater, which the reaction tank is made up of three tanks: anaerobic, anoxic, and aerobic. Many previous studies used the method to remove nitrogen and phosphorus from domestic wastewater [12, 13] and slaughterhouse wastewater [14]. However, a large amount of space is required to construct the system, making it unsuitable for small scale studies. Therefore, using microbes that have been treated directly in wastewater makes this study more feasible without requiring a large amount of experimental space.

The use of microbes to treat hatchery wastewater has received relatively little attention, particularly in the application of mixed culture. Many previous studies have highlighted the numerous benefits of mixed culture over pure culture. Priyadharshini and Kumar [15] discovered that the removal efficiencies for COD mixed culture were 63% compared to 57% for COD removal in pure *Bacillus* sp. culture. While, a study conducted by Tangahu et al. [16] on Batik wastewater treatment using the intermittent method indicated that mixed culture of *Scirpus grossus* and *Iris pseudacorus* removed up to 89% and 97% of COD and BOD, respectively, as compared to a single culture. In other studies, Masser et al. [17] showed that a mixed culture of four species of bacteria removed 69% of total phosphorus from rubber wastewater, which was much greater than when each bacterium was treated alone. Hence, this study attempts to highlight the application of mixed cultures instead of pure culture for the treatment of hatchery wastewater. The goals of this research were to characterize the wastewater and to identify the optimum condition of the biological treatment of contaminated wastewater using acclimatized mixed culture. The optimum condition obtained has the potential to significantly remove nutrients and COD from wastewater by optimizing it using a central composite design (CCD).

## 2 Material and methods

### 2.1 Collection of samples

All samples were taken from a pond located in Setiu, Terengganu, Malaysia. The pond water was collected in order to create synthetic wastewater with a similar composition to the pond wastewater, and the pond sediment was collected to serve as the mixed culture [18]. Store-bought stones with diameters ranging from 1.5 to 2.0 cm were chosen.

### 2.2 Preparation of synthetic wastewater

Synthetic wastewater was formulated in accordance with hatchery wastewater characterization to achieve a COD concentration of 250 mg/L, a nitrate-N concentration of 10 mg/L, and a total phosphorus concentration of 10 mg/L. It was critical to generate synthetic wastewater with precise COD, nitrate-N, and phosphorus concentrations in order to standardize their contents throughout the studies and minimize experimental error. To prepare 10 L synthetic wastewater, 7g of fish food pellet solution (CNS) was mixed with 10 L tap water and autoclaved at 121 °C for 15 min. 250 mL of prepared synthetic wastewater was used to feed mixed cultures daily for 14 days.

### 2.3 Acclimatization of mixed cultures

Acclimatization was accomplished to ensure that the COD content in the sample could be adequately adapted by the mixed culture. A bioreactor was filled with 1250 mL of mixed culture and 3750 mL of nutrient stock [16]. Next, the total solids and COD concentrations were identified, which were 29294 mg/L and 285.67 mg/L, respectively. This procedure was carried out by administering 250 mL of nutritious stock daily for two weeks.

### 2.4 Experimental setup for optimization

Table 1 shows the factors and ranges of the CCD experimental setup. The experiments were developed using the central composite design (CCD) to determine the optimum process parameters of two factors, namely acclimatized mixed culture (AMC) content and retention time, using Response Surface Methodology (RSM). The experimental setup created by Design-Expert software is shown in Table 2. The experiment was conducted in a conical flask accordingly, with a total of 13 experimental runs. Conical flasks with varying concentrations of AMC and synthetic wastewater were filled to a total volume of 200 mL. Samples were kept at room temperature at various retention times. Samples were collected. Chemical oxygen demand (COD), nitrate-N, and total phosphorus levels were determined in samples. The outputs were inserted into the software and analysed with a 95% confidence level using analysis of variance (ANOVA).

**Table 1:** The range of factor values used in central composite design.

Factor	-α	-1	0	+1	+α
A: Acclimatized Mixed Culture (AMC) content	15	20	25	30	35
B: Retention time (days)	3	3.5	4	4.5	5

**Table 2:** Experimental data of CCD.

Std	Factors		Responses		
	A	B	COD	Nitrate-N	Total P
1	20	3.50	26	98	0
2	30	3.50	14	69	36
3	20	4.50	30	56	17
4	30	4.50	50	41	6
5	15	4.00	24	62	0
6	35	4.00	38.8	94	5
7	25	3.00	19	98	0
8	25	5.00	62	62	1.6
9	25	4.00	28	74	40
10	25	4.00	27	75	46
11	25	4.00	31.5	78	42
12	25	4.00	30	72	35
13	25	4.00	31.5	77	38

A: Acclimatized mixed culture (AMC); B: Retention time.

### 2.5 Sample analysis

The analysis of the sample was carried out immediately after collection in accordance with the retention time (Table 2). Chemical oxygen demand (COD), nitrate-N, and total phosphorus were the three responses that were determined.

### 2.6 COD analysis

COD test was performed to identify the initial and final readings used to calculate the removal of COD from the wastewater. In order to calculate the COD, 0th-day sample was used to detect the initial value of COD for all 10 containers in the 10th day. The COD vial was mixed with 2 mL of the 0th-day sample. Blank was prepared by mixing 2 mL of deionized water into the COD vial. Both the sample and blank vials were heated in a DRB200 reactor for 120 min at 150 °C before being tested. The test for the blank and sample was repeated three times to get an average reading using a DR900 Hach spectrophotometer.

#### 2.6.1 Nitrate-N analysis

To calculate nitrate-N, the sample and the blank were prepared by adding 10 ml of the sample. One NitraVer 5 nitrate reagent pillow was added into the sample bottle. The DR900 Hach spectrophotometer was used to test both the sample and the blank.

#### 2.6.2 Total phosphorus analysis

Molybdovanadate with Acid Persulfate Digestion Method (Method 10127) was used to measure total phosphorus. Deionized water was added to the wastewater and acclimatized mixed culture (AMC) mixtures to dilute to dilution factors of five and ten, respectively. The Hach spectrophotometer was then used to determine the total removal of phosphorus.

## 3 Results and discussions

### 3.1 Fitting the model

Table 2 displays the experimental results where, among the center points of 25% AMC and 4 days of retention time, run 11 exhibits the highest percentage removal for COD and nitrate-N. Meanwhile, run 10 had the greatest total phosphorus removal. It is also observed that different parameters contribute to the removal of pollutants differently. The highest removal of COD was observed at 62%, 98% for nitrate-N and 46% for total phosphorus. The analysis of variance demonstrates that COD and total phosphorus are adequately represented by a quadratic polynomial model after being fitted by different models (linear, two-factorial, quadratic and cubic). According to the analyses, the cubic polynomial model was determined to be the best for nitrate-N. The adjusted R^2^ of the quadratic model for COD and total phosphorus (0.9577 and 0.8907, respectively) was the highest among the linear, two factorial and cubic models. In the meantime, the cubic model had the highest adjusted R^2^ when compared to the linear and two factorial models for nitrate-N (0.7366). The quadratic polynomial model was insignificant for nitrate-N.

### 3.2 Statistical analysis for COD

#### 3.2.1 Analysis of variance (ANOVA)

**Figure 1:**
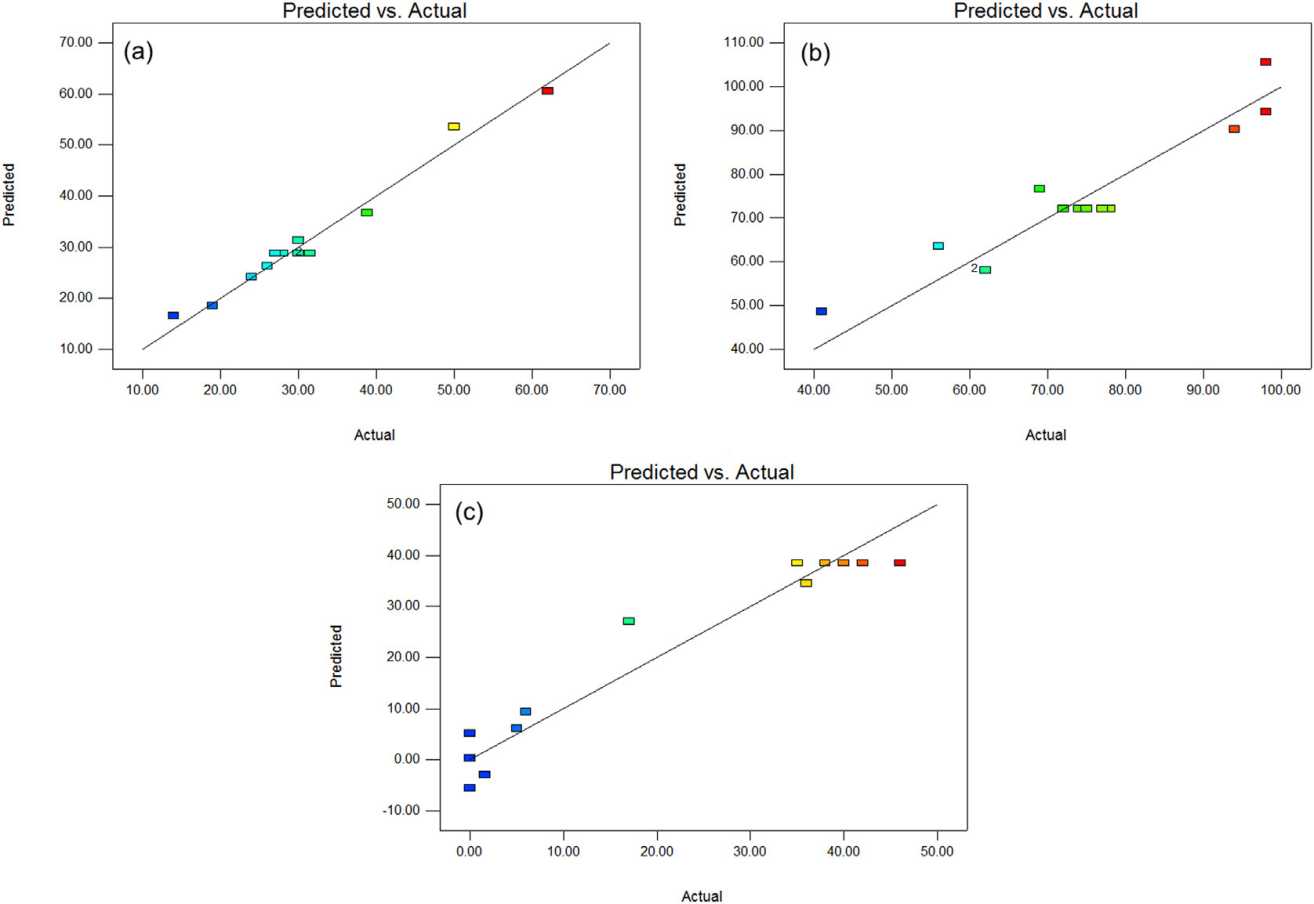
Predicted against actual plot of (a) COD, (b) nitrate-N, and (c) total phosphorus removal.

The ANOVA (F-test) and p-value of COD, which model coefficient is being assessed, and the parameters are examined for significance, are described in Table 3. It is also used to indicate the interaction strength between factors. The confidence level portrayed by the ANOVA table was considerably higher than 95%, while the p-value was much lower than 0.0001. A p-value less than 0.05 indicated that the model was significant, indicating that it was appropriate for this experiment. Both main effects and their interactions were significant based on the p-value of less than 0.05. Meanwhile, the “lack of fit” of this model is not significant with a p-value of 0.2053, indicating the quadratic model is adequate. The R^2^ of 0.9753 and adjusted R^2^ of 0.9577 in which higher than 0.80 signified that the proposed model represents the experimental data well. As mentioned by Lee et al. [19], the R^2^ for a good fit of a model should be at least 0.80, hence this regression model described the process effectively. The predicted against actual data on COD removal is presented in Figure 1a. A linear distribution was seen, indicating that the model fit the data well. The predicted values were in close agreement to the observed values for COD removal. For COD removal, the predicted values were in close agreement to the actual values.

**Table 3:** ANOVA for COD removal response surface model.

Source	Sum of squares	Mean square	F value	P-value Prob > F	Level of significance
Model	1864.23	372.85	55.35	<0.0001	Significant
A-AMC Content	117.81	117.81	17.49	0.0041	
B-Retention time	1323.00	1323.00	196.42	<0.0001	
AB	256.00	256.00	38.01	0.0005	
A^2^	3.71	3.71	0.55	0.4824	
B^2^	164.23	164.23	24.38	0.0017	
Residual	47.15	6.74			
Lack of Fit	30.45	10.15	2.43	0.2053	Not significant
Cor Total	1911.38				
R^2^	0.9753				
R_adj_	0.9577

#### 3.2.2 Effect of AMC content and retention time on COD removal

**Figure 2:**
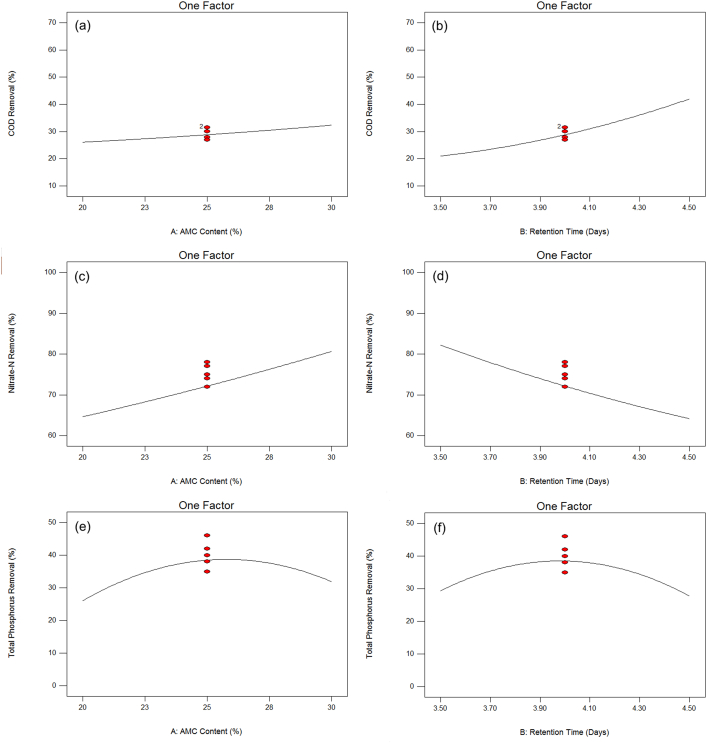
Effect of AMC content on, % (a) COD, (c) nitrate-N, and (e) total phosphorus removal, and effect of retention time, days on (b) COD, (d) nitrate-N, and (f) total phosphorus removal.

Figure 2 depicts the influence of AMC content and retention time on COD removal. Figure 2a shows that increasing the AMC content from 20% to 30% resulted in an increase in COD removal. Similarly, increasing the retention time from 3.5 days to 4.5 days resulted in an increase in COD removal, as shown in Figure 2b. Oljira et al. [20] had demonstrated that the removal of COD in the brewery effluents increases with the increase in incubation period. Moreover, instead of a single culture application, the combination of three different bacteria species showed a remarkable increment in the removal of COD. The treatment with the consortium of *Aeromonas* sp., *Pseudomonas* sp., and *Bacillus* sp. gave rise to a 93.25% removal efficiency of COD, as opposed to the individual treatments of 76.78%, 79.61%, and 82.67% removal, respectively. The organic material presents in the wastewater operates as a substrate for the aerobic microbial metabolism, which explains the reduction of COD concentration. The removal efficiency increased as the incubation period lengthened. Their results were consistent with this study, which found that the rate of COD removal increased over time and that the bacterial content is critical to the process of improving removal.

**Figure 3:**
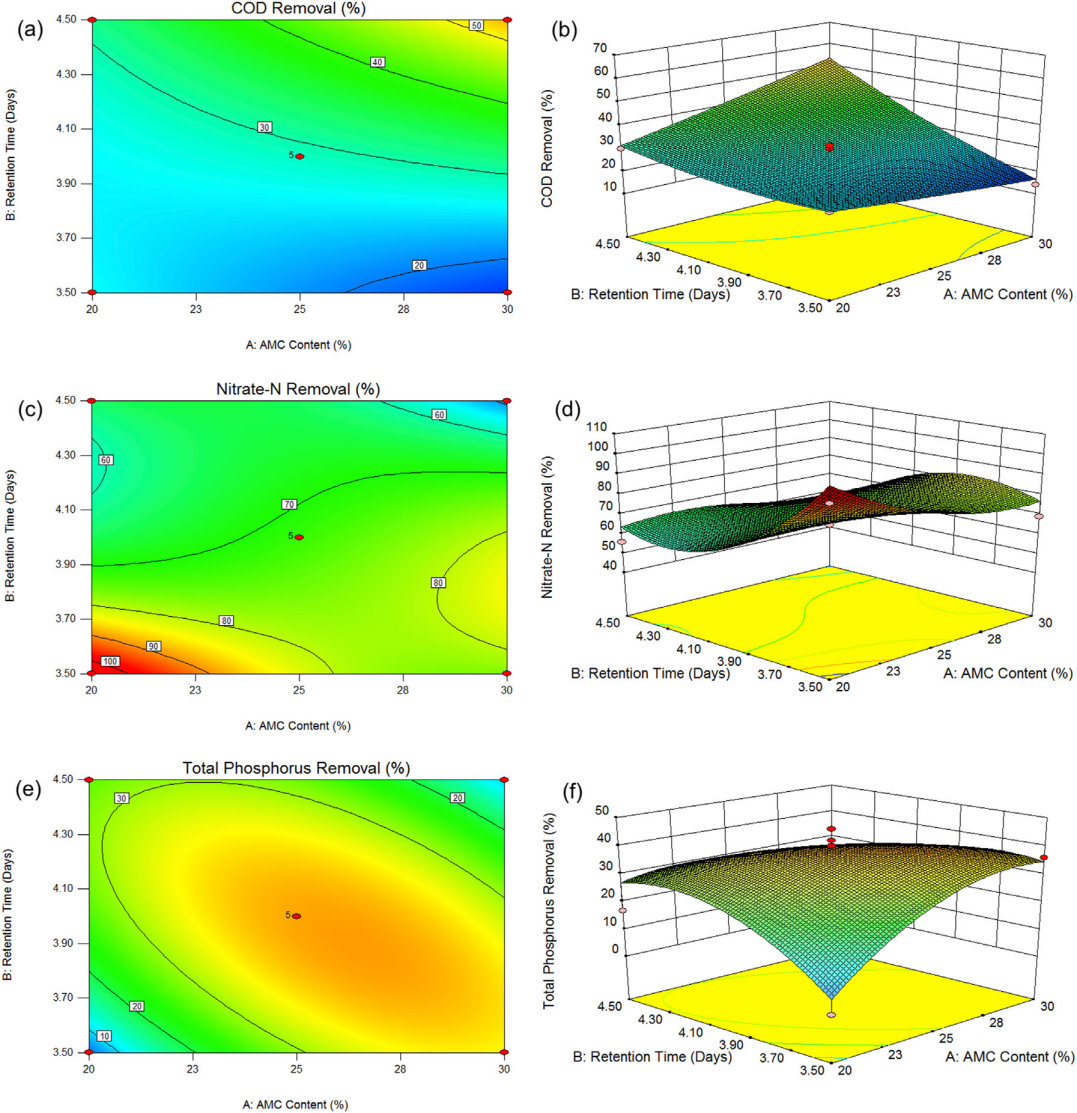
Contour plots of (a) COD, (c) nitrate-N, and (e) total phosphorus removal, and 3D response surface plots of (b) COD, (d) nitrate-N, and (f) total phosphorus removal as function of AMC content and retention time.

The influence of AMC content and retention time on COD removal is shown in Figure 3. The plot demonstrated that the retention time had an effect on COD removal for any AMC content. The amount of COD removal increased as the retention time was increased.

### 3.3 Statistical analysis for Nitrate-N

#### 3.3.1 Analysis of variance (ANOVA)

The ANOVA for nitrate-N was presented in Table 4. It was found that the model had a p-value of 0.0354, which was less than 0.05, indicating that it was statistically significant. The p-value for both main effects was lower than 0.05 and therefore was significance; meanwhile, the interaction effect for nitrate-N was insignificant, proved by its p-value of 0.4485, which was higher than 0.05. Meanwhile, the “lack of fit” of this model is significant with a p-value of 0.0015, indicating the quadratic model is not adequate. The R^2^ value of 0.8903 explains that the estimated model suits the experimental data adequately. The predicted versus actual plot on nitrate-N removal was presented in Figure 1b. The linear distribution showed that the model was fitted well, and that the values of predicted and observed were in good agreement.

**Table 4:** ANOVA for nitrate-N removal.

Source	Sum of squares	Mean square	F value	P value Prob > F	Level of significance
Model	2942.54	420.36	5.80	0.0354	Significant
A-AMC Content	512.00	512.00	7.06	0.0451	
B-Retention time	648.00	648.00	8.93	0.0305	
AB	49.00	49.00	0.68	0.4485	
A^2^	5.93	5.93	0.082	0.7864	
B^2^	23.31	23.31	0.32	0.5953	
Residual	362.69	72.54			
Lack of Fit	339.89	339.89	59.63	0.0015	Significant
Cor Total	3305.23				
R^2^	0.8903				
R_adj_	0.7366				

#### 3.3.2 Effect of independent and interactive parameters on nitrate-N removal

Figure 2 depicts the influence of two independent parameters on nitrate-N removal. As shown in Figure 2c, the removal of nitrate-N was increased with the increase in AMC content, while an increase in retention time caused the decrease in nitrate-N removal, as depicted in Figure 2d. These suggest that three days were sufficient for the microbes to remove nitrate-N, as the maximum removal of nitrate-N was observed on day 3. Nitrate was required for the growth of the mixed culture. Increased retention time could promote the growth of bacteria that digest all impurities, allowing the wastewater to be cleansed [21]. Nitrate removal via the denitrification process requires an ample amount of oxygen [22, 23]. Therefore, oxygen was reduced as a result of the longer retention time, which eventually diminished the removal of nitrate. Lu et al. [24] in their research found that 0.7 h was the optimum hydraulic retention time instead of 12 h for the removal of nitrogen. Furthermore, the nitrite oxidation activity was significantly increased with a decreased hydraulic retention time from 30 to 5 h. This is due to the increase in nitrite-oxidizing bacteria, where the short hydraulic retention time favoured the relative growth of these bacteria, particularly the fast-growing *Nitrobacter* sp [25]. As pointed out by Oljira et al. [20], the mixed combination of *Aeromonas* sp., *Pseudomonas* sp., and *Bacillus* sp., had shown a significant contribution to the removal of total nitrogen, as compared to their single treatment. The synergistic effect on the removal of the pollutants by these three isolates might explain this condition.

The contour plot and 3D response surface were presented in Figures 3c and 3d, representing the effect of interactions between parameters on nitrate-N removal. The optimum removal of nitrate-N was up to 98% at a condition of 20% AMC content and 3.5 days of retention time. An increase in retention time was seen to unnecessarily affect the removal of nitrate-N. Also, it was remarked that the removal of nitrate-N at any AMC content was affected by the retention time.

### 3.4 Statistical analysis for total phosphorus

#### 3.4.1 Analysis of variance (ANOVA)

Table 5 presents the summary of ANOVA for total phosphorus removal. The p-value of less than 0.05, which is 0.0005 was an indication of the significance of the model. Both the main effects of AMC content and retention time displayed an insignificant effect with p-values of 0.1518 and 0.6660, respectively. However, the interaction effect was statistically significant at 0.0073. Meanwhile, the “lack of fit” of this model is not significant with a p-value of 0.1062, indicating the quadratic model is adequate. Both R^2^ and adjusted R^2^ of 0.9362 and 0.8907, respectively, demonstrated the estimated model adequately fits the experimental data. Figure 1c portrays the predicted against the actual plot for total phosphorus removal. Again, as seen in all responses, the linear distribution was observed, indicating that the model was well-fitted. The values of predicted and observed were in close agreement.

**Table 5:** ANOVA for total phosphorus removal.

Source	Sum of squares	Mean square	F value	P-value Prob > F	Level of significance
Model	4054.03	810.81	20.55	0.0005	Significant
A-AMC Content	102.08	102.08	2.59	0.1518	
B-Retention time	8.00	8.00	0.20	0.6660	
AB	552.25	552.25	14.00	0.0073	
A^2^	2081.67	2081.67	52.76	0.0002	
B^2^	2271.45	2271.45	57.57	0.0001	
Residual	276.18	39.45			
Lack of Fit	207.38	69.13	4.02	0.1062	Not significant
Cor Total	4330.21				
R^2^	0.9362				
R_adj_	0.8907				

#### 3.4.2 Effect of AMC content and retention time on total phosphorus removal

The influence of AMC content and retention time on total phosphorus is depicted in Figure 2. There was an increase in the removal of phosphorus with the increase in AMC content until a maximum value of 46% at the centre point and was decreased after reaching the maximum value (Figure 2e). A comparable patent was also seen for retention time, represented by Figure 2f where the removal of total phosphorus was increased with the increase of retention time and achieved its maximum value at the centre point. Naili et al. [26] mentioned that the mixed culture of *Alcaligenes denitrificans, Moraxella lacunata*, *Pseudomonas aeruginosa*, and *Acinetobacter junii* had contributed significantly to the removal of phosphate in the wastewater and that the synergistic relationship between the co-cultured species had accelerated the removal process.

The influence of interaction between AMC content and retention time was graphically illustrated by Figures 3e and 3f using contour plot and 3D response, respectively. The presence of a significant interaction between the parameters was indicated by an elliptical contour plot. It was noticed from the figure that the optimum removal of total phosphorus was obtained at 25% AMC content and at 4 days of retention time.

### 3.5 Optimum condition

Table 6 displays the optimum conditions suggested by the Design-Expert software obtained from the analysis of RSM. It was suggested that at AMC content of 29% with a retention time of 4 days, as much as 28%, 80%, and 36% of COD, nitrate-N and total phosphorus removal could be obtained. At 25% of AMC content and 4 days of retention time, the highest COD, nitrate-N and total phosphorus measured were 31.5%, 78% and 42% respectively. However, to achieve maximum removal for each response, the conditions were seen varied among responses. The removal of COD was up to 62% in a condition of 25% AMC content and 5 days of retention time. For nitrate-N, at 25% AMC content and 3 days of retention time had given rise to the removal value of up to 94%. To reach the maximal removal of 46% for total phosphorus, the optimal condition was at 25% of AMC content and 4 days of retention time. All responses demonstrated the same AMC content of 25% for the maximum removal with varied retention times.

**Table 6:** Suggested optimum conditions.

Factor	Value
A: AMC content	29 %
B: Retention time	4 days
COD removal	28 %
Nitrate-N removal	80 %
Total phosphorus removal	36 %

## 4 Conclusion

This study focused on the application of acclimatized mixed culture (AMC) in removing chemical oxygen demand (COD), nitrate-N and total phosphorus. The conditions of 29% AMC content and a retention time of 4 days were optimum for the removal process as determined by central composite design, with a maximum removal of 31.5% COD, 78% nitrate-N, and 46% total phosphorus obtained. While separating conditions to achieve the maximum removal value resulted in 62%, 94%, and 46% removal of COD, nitrate-N, and total phosphorus, respectively. The results demonstrate that acclimatized mixed culture can naturally remove COD, nitrate-N and total phosphorus.

## Declarations

### Author contribution statement

Norazwina Zainol: Conceived and designed the experiments; Contributed reagents, materials, analysis tools or data.

Kamaliah Abdul Samad: Analyzed and interpreted the data; Wrote the paper.

Che Asilah Ilyana Che Jazlan: Conceived and designed the experiments; Performed the experiments; Analyzed and interpreted the data; Wrote the paper.

Nurul Ain Razahazizi: Conceived and designed the experiments; Performed the experiments.

### Funding statement

This work was supported by Universiti Malaysia Pahang (RDU210341).

### Data availability statement

The data that has been used is confidential.

### Declaration of interests statement

The authors declare no conflict of interest.

### Additional information

No additional information is available for this paper.
